# Hygrolansamycins A-D, *O*-Heterocyclic Macrolides from *Streptomyces* sp. KCB17JA11

**DOI:** 10.4014/jmb.2206.06039

**Published:** 2022-09-05

**Authors:** Jun-Pil Jang, Byeongsan Lee, Kyung Taek Heo, Tae Hoon Oh, Hyeok-Won Lee, Sung-Kyun Ko, Bang Yeon Hwang, Jae-Hyuk Jang, Young-Soo Hong

**Affiliations:** 1 Chemical Biology Research Center, Korea Research Institute of Bioscience and Biotechnology, Cheongju 28116, Republic of Korea; 2 College of Pharmacy, Chungbuk National University, Cheongju 28160, Republic of Korea; 3 KRIBB School of Bioscience, University of Science and Technology, Daejeon 34141, Republic of Korea; 4 Biotechnology Process Engineering Center, Korea Research Institute of Bioscience and Biotechnology, Cheongju 28116, Republic of Korea

**Keywords:** Molecular networking, *Streptomyces*, ansamycin family, hygrolansamycin

## Abstract

Six ansamycin derivatives were isolated from the culture broth of *Streptomyces* sp. KCB17JA11, including four new hygrolansamycins A-D (1-4) and known congeners divergolide O (5) and hygrocin C (6). Compounds 1-5 featured an unusual six-membered *O*-heterocyclic moiety. The isolation workflow was guided by a Molecular Networking-based dereplication strategy. The structures of 1-4 were elucidated using NMR and HRESIMS experiments, and the absolute configuration was established by the Mosher’s method. Compound 2 exhibited mild cytotoxicity against five cancer cell lines with IC_50_ values ranging from 24.60 ± 3.37 μM to 49.93 ± 4.52 μM.

## Introduction

Actinomycetes are the primary producers of ansamycins, including rifamycin, ansamitosin, and geldanamycin, which have potent antibiotic and anticancer properties [1, 2]. In general, 3-amino-5-hydroxybenzoic acid (AHBA) is utilized for the polyketides assembly of ansamycins containing a benzene- or naphthalene-based mC7N chromophore [1]. It has recently been reported that modified ansamycin scaffolds can be generated through divergent biosynthetic pathways involving divergolides and hygrocins [3-7]. Baeyer-Villiger oxygenation of a macrolactam intermediate has also been proposed as a biosynthetic route to the divergolides and hygrocins [6, 8, 9]. Furthermore, several divergolides undergo optional acyl migration following oxidative β-hydroxy formation adjacent to the ester bond [4, 6].

Tandem mass-based dereplication is a common technique for screening known bioactive compounds, such as ansamycins, in complex microbial samples [10-16]. Global Natural Products Social Molecular Networking (GNPS), an open-access data-driven tandem mass spectral platform, is particularly well-suited for this purpose and is widely used. Previously, during our tandem-mass-based dereplication study of *Streptomyces* species, we discovered geldanamycin [10] and streptimidone [11] derivatives. In this study, we investigated the fermentation of *Streptomyces* sp. KCB17JA11, a geldanamycin producer, and using the GNPS system, we discovered new nodes, corresponding to divergolide O (**5**) [7] and hygrocin C (**6**) [5] with molecular weights of 513 and 509, respectively. Four other compounds were found to have the same molecular formula (C_31_H_39_NO_8_) as divergolide O (**5**). Herein, we present four new stereochemical derivatives of **5**, namely hygrolansamycins A-D (**1-4**).

## Materials and Methods

### General Experimental Procedures.

Optical rotation was obtained using a JASCO P-1020 polarimeter. UV spectra were recorded on an Optizen 2120 UV spectrophotometer. NMR experiments were operated on a Bruker AVANCE HD 800 MHz NMR spectrometer (Bruker, Germany) at the Korea Basic Science Institute (KBSI) in Ochang, Korea. NMR spectra were recorded in DMSO-d_6_ as an internal standard (δ_H_ 2.50/δ_C_ 39.51). High resolution electrospray ionization mass spectra (HR-ESIMS) were recorded on a Waters Vion IM-QTOF mass spectrometer (Waters, USA) at the KRIBB in Ochang, Korea. Column chromatography was performed on reversed phase silica gel (0.075 mm; Cosmosil, Japan). Analytical C_18_ (Waters Sunfire, 5 μm, 4.6 × 150 mm) and semi-preparative C_18_ (Waters Atlantis T3, 5μm, 10 × 250 mm) columns were used for reverse phase HPLC on a 515 pump HPLC system (Waters) equipped with a 2996 PDA detector (Waters) using HPLC grade solvents (Honeywell). Liquid chromatography-mass spectrometry (LC-MS) was operated using an LTQ XL linear ion trap (Thermo Scientific, USA) equipped with an electrospray ionization (ESI) source that was coupled to a rapid separation LC (RSLC; ultimate 3000, Thermo Scientific) system (ESI-LC-MS).

### Strain Identification

A soil sample was collected at Ochang, Cheongju, Republic of Korea. Analysis of 16S rRNA gene sequences showed that strain KCB17JA11 was most closely related to the *Streptomyces rapamycinicus* gene (99.79% identity, GenBank Accession No. KP209440.1). Therefore, strain KCB17JA11 was named *Streptomyces* sp. KCB17JA11 and used in the subsequent culture experiments.

### Culture Conditions

*Streptomyces* sp. KCB17JA11 was cultured for 3 days at 28°C on a rotary shaker with agitation at 125 rpm in a 250 ml Erlenmeyer flask containing 50 ml of seed culture medium (soluble starch 1%, yeast extract 0.1%, and tryptone 0.1%). For a large culture,1% of the pre-culture broth was inoculated into 40 × 1,000 ml baffled Erlenmeyer flasks containing 250 ml of YMG broth (glucose 1%, soluble starch 2%, meat extract 0.3%, yeast extract 0.5%, malt extract 0.5%, and CaCO_3_ 0.05%), and cultured for 8 days at 28°C on a rotary shaker with agitation at 125 rpm.

### LC-MS Analysis Conditions and Dereplication through Molecular Networking

The samples from the EtOAc extract were dissolved in a methanol and analyzed using a Thermo U3000-LTQ XL ion trap mass spectrometer (Thermo Scientific) equipped with an electrospray ionization (ESI) mass source. Chromatographic separation of the compounds was achieved using a Waters HSS T3 C_18_ column (2.1 × 150 mm; 2.5 μm) at a flow rate of 0.3 ml/min. The mobile phases A and B were water and acetonitrile, respectively, both containing 0.1% formic acid. Gradient elution was conducted as follows: 5–100% B for 0–15 min with a linear gradient, followed by 5 min of 100% B. The MS/MS system was operated in ESI mode. Typical operating parameters were as follows: spray needle voltage, +5 kV; ion transfer capillary temperature, 275°C; nitrogen sheath gas, 35; and auxiliary gas, 5 (arbitrary units). The ion trap contained helium damping gas which was introduced in accordance with the manufacturer’s recommendations. Mass spectra were acquired in an *m/z* range of 100–2,000, applying three microscans and a maximum ion injection time of 100 ms. Data-dependent mass spectrometry experiments were controlled using the menu-driven software provided in the Xcalibur system (version 4.0; Thermo Scientific). The acquired data file was exported in *.mzXML format using the application of MS-convert software, which is a part of the ProteoWizard package. Molecular networks were generated using Global Natural Products Social Molecular Networking (http://gnps.ucsd.edu). The parameters were set as follows: product ion tolerance of 0.5 Da and a precursor ion mass tolerance of 2.0 Da. The results were visualized using Cytoscape 3.7.2 software.

### Extraction and Isolation

To remove EtOAc, the residue was partitioned three times with EtOAc and evaporated. The crude extract was fractionated employinng reversed-phase C_18_ vaccum column chromatography eluting with a stepwise MeOH:H_2_O solvent system of (20: 80 to 100: 0, each × 1 L). The 70% (1,043.4 mg) fraction was further fractionated using a CombiFlash RF (Teledyne ISCO) medium-pressure chromatography system (MPLC) on a Redisep RF C_18_ reverse-phase column under stepwise gradient elution with MeOH-H_2_O (from 20: 80, 40: 60, 60: 40, 80: 20 to 100: 0; 1 L for each step). To obtain compound **6**, fraction 3 (344 mg) was subjected to semi-preparative HPLC [Waters Atlantis T3 C_18_ column (10 × 250 mm, 5 μm, 3 ml/min)] under isocratic elution using 65% MeOH-H_2_O (0.05%TFA) over 45 min to (6.0 mg, RT:16.1). The same MPLC fraction 3 was subjected to semipreparative HPLC [Waters Atlantis T3 C_18_ column (10 × 250 mm, 5 μm, 3 ml/min)] applying gradient elution of 50% CH_3_CN-H_2_O (0.05% TFA) over 35 min to yield compounds **1** (29.0 mg, *t*_R_ 17.5 min), **2** (12.5 mg, *t*_R_ 18.3 min), **3** (6.0 mg, *t*_R_ 19.5 min), **4** (5.0 mg, *t*_R_ 19.1 min), and **5** (11.9 mg, *t*_R_ 15.2 min) respectively.

*Hygrolansamycin A *(**1**). Pale yellow powder; ${\left[\text{α}\right]}_{\text{D}}^{\text{25}}$+43.6 (*c* 0.1, MeOH); UV (MeOH) λ_max_ (log ε) 210 (4.54), 308 (2.92); ^1^H and ^13^C NMR data, Table 1; HRESIMS *m/z* 512.2285 [M -H]^-^ (calcd for C_28_H_35_NO_8_, 512.2289 (Fig. S20)).

*Hygrolansamycin B* (**2**). Pale yellow powder; ${\left[\text{α}\right]}_{\text{D}}^{\text{25}}$+22.6 (*c* 0.1, MeOH); UV (MeOH) λ_max_ (log ε) 208 (4.50), 309 (2.90); ^1^H and ^13^C NMR data, Table 1; HRESIMS *m/z* 512.2285 [M -H]^-^ (calcd for C_28_H_35_NO_8_, 512.2289 (Fig. S29)).

*Hygrolansamycin C* (**3**). Pale yellow powder; ${\left[\text{α}\right]}_{\text{D}}^{\text{25}}$-12.82 (*c* 0.1, MeOH); UV (MeOH) λ_max_ (log ε) 210 (4.40), 310 (2.80); ^1^H and ^13^C NMR data, Table 1; HRESIMS *m/z* 512.2285 [M -H]^-^ (calcd for C_28_H_35_NO_8_, 512.2289 (Fig. S38)).

*Hygrolansamycin D* (**4**). Pale yellow powder; ${\left[\text{α}\right]}_{\text{D}}^{\text{25}}$+52.2 (*c* 0.1, MeOH); UV (MeOH) λ_max_ (log ε) 210 (4.48), 308 (2.82); ^1^H and ^13^C NMR data, Table 1; HRESIMS *m/z* 512.2285 [M -H]^-^ (calcd for C_28_H_35_NO_8_, 512.2289 (Fig. S11)).

### Modified Mosher’s Method

Compounds **1** and **4** (0.5 mg) was dissolved in anhydrous pyridine (1 ml), and a catalytic amount of dimethylaminopyridine (DMAP) was added. After 5 min of stirring, 25 μl of (*R*)-MPA-Cl was added, and the mixture was stirred at room temperature for 16 h. Repeat treatment of same method with (*S*)-MPA-Cl instead of (*R*)-MPA-Cl. Each mixture was subjected to semi-preparative reversed phase HPLC (column as above; flow rate 3 ml/min; 50-100% CH_3_CN−H_2_O containing 0.05% TFA over 25 min) to yield (*S*)-MPA ester **1a** (0.3 mg, *t*_R_ 16.5 min) and **4a** (0.3 mg, *t*_R_ 16.8 min) and (*R*)-MPA ester **1b** (0.3 mg, *t*_R_ 17.0 min) and **4b** (0.3 mg, *t*_R_ 17.3 min). The ^1^H NMR chemical shifts of MPA esters (**1a, 1b, 4a**, and **4b**) were assigned on the basis of interpretation of ^1^H, COSY, and HSQC-DEPT NMR data (see Supporting Information for NMR data).

### Cell Viability Assay

B16F10, HeLa, MDA-MB-231, and PC12 cell lines were grown in Dulbecco’s modified Eagle’s medium (DMEM; Welgene, LM 001-05) supplemented with 10% fetal bovine serum (FBS; Welgene, S001-07), 100 units/ml penicillin, and 100 μg/ml streptomycin (Gibco, 15140-122) in a humidified atmosphere at 37°C with 5% CO_2_. The AGS cells were grown in Roswell Park Memorial Institute 1640 (RPMI 1640; Welgene, LM 011-01) medium supplemented with 10% FBS, penicillin, and streptomycin. Cells were seeded in 96-well cell culture plates (0.7 × 10^4^ cells/well) overnight. Varying concentrations of compounds were treated for 24 h, and 10 μl of EZ-Cytox cell viability assay solution (Daeil Lab Service, Korea) was directly added. After 2 h of incubation at 37°C, the absorbance was measured at 450 nm using a microplate reader (Molecular Devices, USA).

## Results and Discussion

**Fig. 1:**
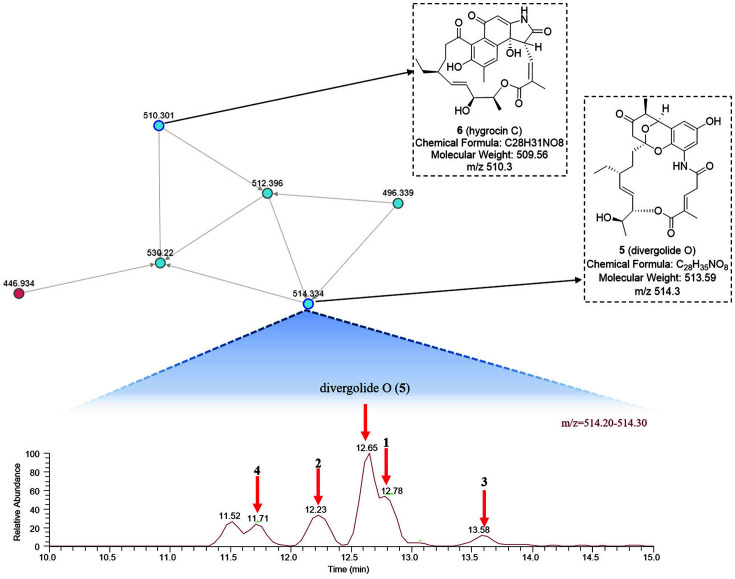
Molecular network of metabolites produced by *Streptomyces* sp. KCB17JA11. Nodes are connected if the cosine similarity of fragment spectra is ≥0.7. The upper node shows molecular networks connected to divergolide O (**5**). In the same node, there were five additional precursor mass peaks, *m/z* 514.

**Fig. 2:**
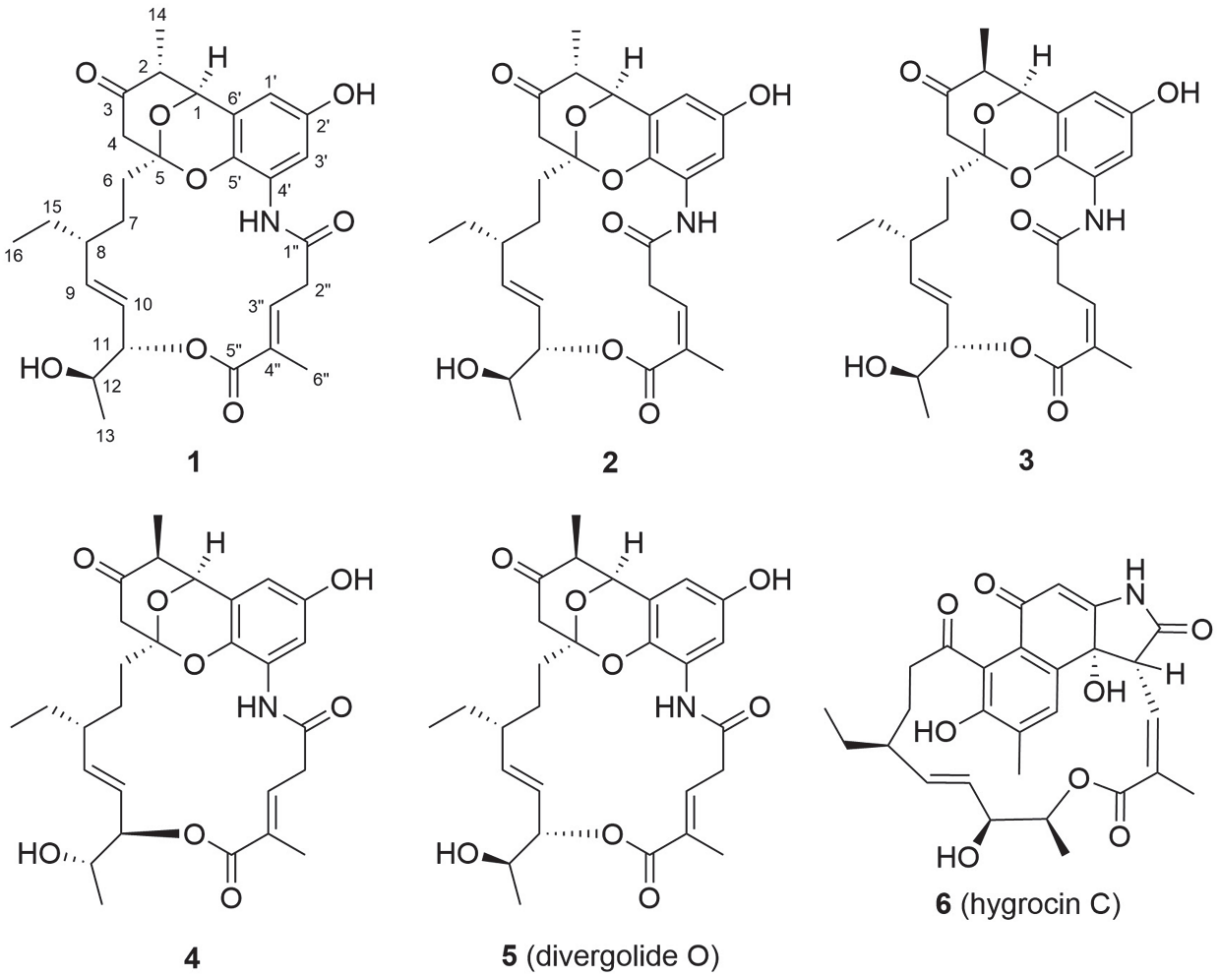
Chemical structures of hygrolansamycins A-D (1-4), divergolide O (5), and hygrocin C (6).

*Streptomyces* sp. KCB17JA11 was cultured in YMG media at 28°C for 7 days, before the broth and mycelia extracts were partitioned using EtOAc. The EtOAc extracts were examined using ESI-ion trap-MS/MS and the data were processed into molecular networks using the GNPS platform (http://gnps.ucsd.edu) [13]. Among the nodes, the node shown in Fig. 1 was annotated through library searching, corresponding to divergolide O (**5**) and hygrocin C (**6**). The precursor ion at this node had an *m/z* value of 514.3 [M+H]^+^. The High-performance liquid chromatography (HPLC) data and total ion current of the crude extract revealed the presence of six peaks eluting at 11.52, 11.71, 12.23, 12.65, 12.78, and 13.58 min, all of which had the same *m/z* values (514.3 [M+H]^+^) as the standard **5**, which eluted at 12.65 min, as confirmed by retention time and MS/MS spectra comparison (Fig. 1). To obtain compounds **1-5**, purification was performed using silica open-column liquid chromatography, reverse-phase MPLC, and semi-preparative HPLC. Thus, five compounds had *m/z* values of 514.3. Furthermore, **6** was isolated and identified from the same crude extract fractions (Fig. 2).

Hygrolansamycin A (**1**) was obtained as a yellow powder. Based on high resolution electrospray ionisation mass spectroscopy (HRESIMS) data, the molecular formula of **1** was assigned as C_28_H_35_NO_8_, having 12 degrees of unsaturation. The ^1^H NMR spectrum of data of **1** contained signals for five sp^2^ protons (δ_H_ 7.51, 7.00, 6.22, 5.63, and 5.30), three sp^3^ oxymethine protons (δ_H_ 5.04, 5.02, and 3.72), one methyl singlet (δ_H_ 1.86), two methyl doublets (δ_H_ 1.08 and 1.28), and one methyl triplet (δ_H_ 0.83). The ^13^C NMR was combined with HSQC-DEPT data, which indicated resonances for one ketone carbonyl (δ_C_ 208.3), two ester/amide carbonyls (δ_C_ 166.6, 166.4), ten methines including five olefinic methines (δ_C_ 136.3, 133.4, 128.0, 106.6, and 107.7) and three oxygenated methines (δ_C_ 77.3, 75.4, and 68.5), five methylenes (δ_C_ 48.5, 38.1, 37.5, 28.8, and 27.9), and four methyl groups (δ_C_ 19.2, 13.1, 12.4, and 15.5) (Table 1).

**Fig. 3:**
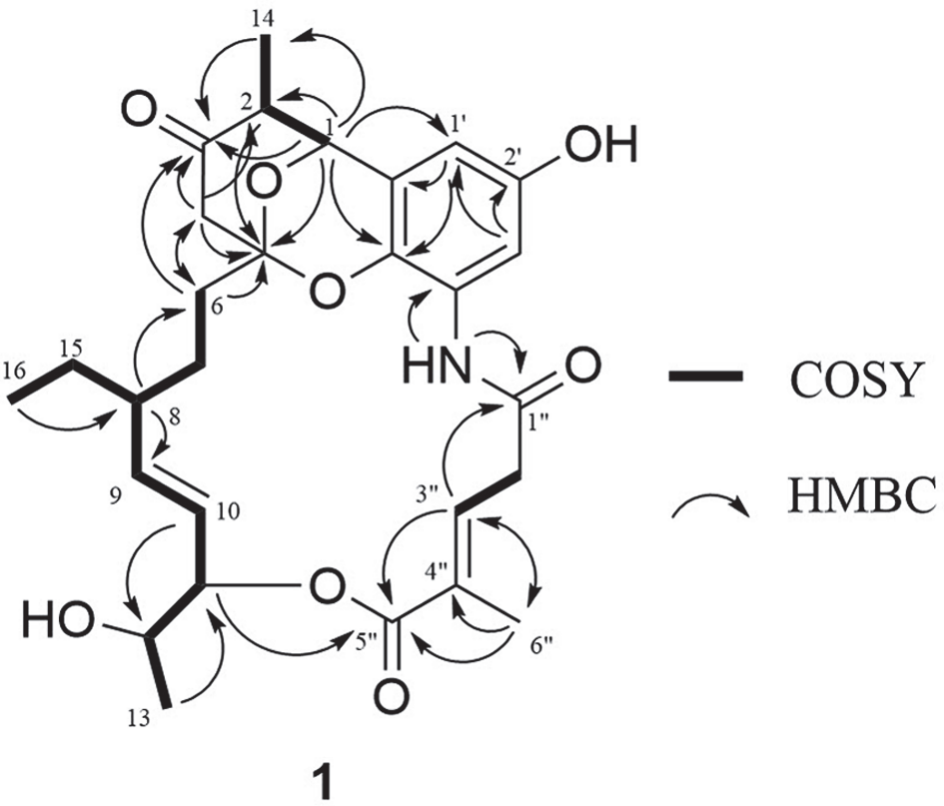
Key 2D NMR correlations of compound 1.

**Fig. 4:**
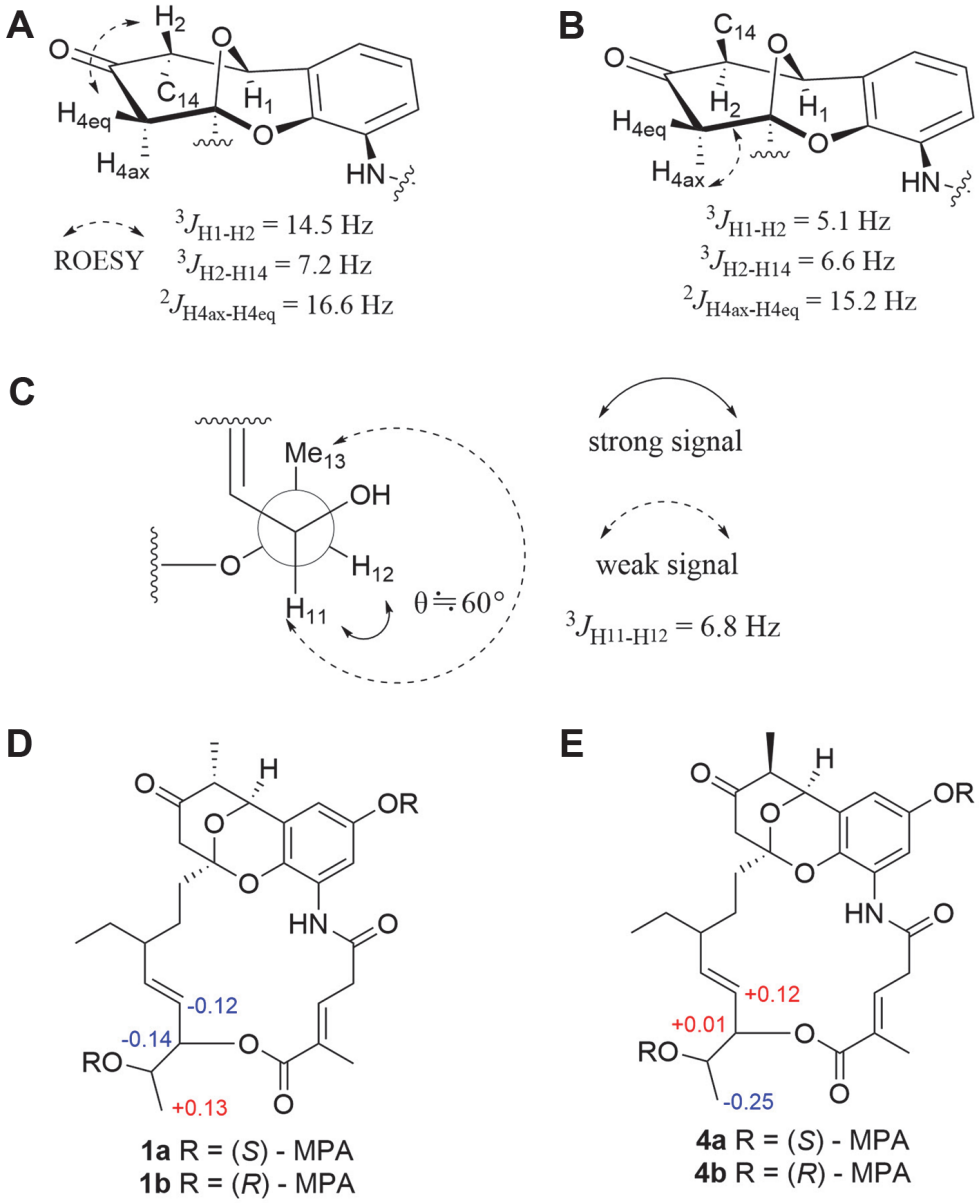
Determination of stereochemistry of compounds. The key coupling constants and ROESY correlations from H-2 to H-4_eq_ or H-4_ax_ in *O*-heterocyclic ring moiety of **1** (A) and **3** (B). The key ROESY correlations and dihedral angle model for determining the relative configurations of C-11 and C-12 in **1** (C). Modified Mosher's ester analysis of **1** (D) and **4** (E). ΔδS-R value of ^1^H for (*S*)- and (*R*)-MPA esters.

**Fig. 5:**
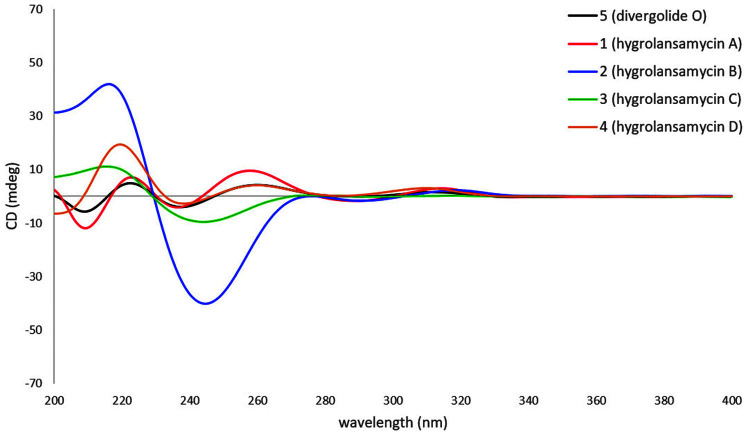
Comparison of the experimental CD spectra of 1-5 in MeOH.

The ¹H-¹H correlation spectroscopy (COSY) spectrum, combined with Heteronuclear multiple bond correlation (HMBC) correlation analysis, established the presence of two spin systems; thus, fragments C-6/C-7/C-8(C-15/C-16)/C-9/C-10/C-11/C-12(C-13) and C-2′′/C-3′′ were assigned. Key correlations from H-11 to C-5′′, H-3′′ to C-5′′ and C-6′′, and H-6′′ to C-3′′, C-4′′, and C-5′′ indicated the presence of two fragments linked via the ester moiety and the methylated position at C-6′′. Furthermore, six typical aromatic carbon signals at δ_C_ 106.6 (C-1′), 151.2 (C-2′), 107.7 (C-3′), 126.4 (C-4′), 132.6 (C-5′) and 122.5 (C-6′), as well as HMBC correlations of NH/C-4′and 1′′, H-3′/C-1′ and C-2′, H-1′/C-5′ and C-6′, suggest the presence of the 1-amino-3-hydroxybenzene moiety. Further analysis of HMBC correlations from H-1 to C-2, C-14, C-3, C-5, and C-5′, from H-4 to C-2, C-3, C-5, and C-6 and from H-6 to C-3, C-4 and C-5 suggested that compound **1** is an unusual six-membered *O*-heterocyclic ring (Fig. 3). Comparison of the planar structural data with those reported for divergolide O (**5**) revealed that 1 was closely related to **5**, except for only relative configurations from H-2 to H-4 rotating-frame nuclear Overhauser effect correlation spectroscopy (ROESY) correlation (Fig. 2). In the previous report, the stereochemical study of *O*-heterocyclic ring moiety had been performed by NMR spectral analysis and X-ray crystallography in divergolide A [4]. In order to establish the relative configuration of *O*-heterocyclic ring moiety of compound 1, detailed analyses of ^1^H NMR and ROESY data were performed. A large coupling constant (^3^J = 14.5 Hz) between H-1 and H-2 showed the anti orientation for the two protons, whereas H-2 and H-14 was showed simply three bond coupling constant (^3^J = 7.2 Hz). The ROESY correlation between H-2 (δ_H_ 2.45) and H-4eq (δ_H_ 2.12) of **1** indicate that equatorial preferred for both H-2 and H-4_eq_ (Fig. 4A). On the other hand, the relative stereochemistry of the *O*-heterocyclic ring moiety in reported divergolide O (**5**) was descripted through NOE correlation between H-2 and H-4_ax_ [7]. In addition, the configuration of the C-3′′/4′′ double bond was assigned as E configuration because of the chemical shift of the allylic methyl group C-6′′ (δ_C_ 13.1) [4]. The absolute configuration of C-11 was determine using a modified Mosher’s method. Methoxyphenylacetic (MPA) esters were prepared by treating **1** with (*R*)- and (*S*)-MPA-Cl in anhydrous pyridine, yielding the corresponding (*S*)- and (*R*)-MPA esters **1a** and **1b**. C-11S configuration was established for **1** based on the Δδ [δ(S) - δ(R)] values of the MPA esters (Fig. 4D). Furthermore, the coupling constant of J_H11/H12_ = 6.8 Hz in **1** indicate that the dihedral angle of these two protons was approximately 60° (Fig. 4C). In addition, the strong ROESY correlation between H-11 and H-12, but not between H-11 and the H-13 methyl protons of **1**, indicated that H-11 was oriented **anti** to this methyl group (Fig. 4C). Thus, the relative configuration of C-1/C-2/C-5/C-11/C-12 was designated as 1S*/2S*/ 5S*/11S*/12S*. However, the relative configuration C-8 bearing the ethylated branch remained unknown because of the lack of relevant ROESY correlations, which was attributed to the flexibility of that portion of the ansa macrolactam. To confirm the configuration of C-8 stereocenter in **1**, using chiroptical analysis involving circular dichroism (CD) spectroscopy. The previously reported CD spectra of **5** and divergolides were elucidated as 1*S*/2R/5*S*/8R/11*S*/12*S* by contrasting with the mirror image and confirming a closely similar Cotton effect to divergolide A [4, 6, 7]. Further, the experimental CD spectra of **1** show strikingly similar Cotton effect from 5 over the entire wavelength range (Fig. 5). Therefore, the absolute configuration of **1** was established 1S/2S/5S/8R/11S/12S. Thus, compound **1** was named as hygorlansamycin A.

Hygrolansamycin B (**2**) was isolated as a yellow powder. According to HRESIMS data the molecular formula of **2** was determined to be C_28_H_35_NO_8_, which is the same as that of **1**. The orientation of the C-3′′/4′′ double bond was the only difference between the structures of **1** and **2**. The relative downfield shift of the C-6′′ allylic methyl group (δ_C_ 13.1, δ_H_ 1.86 in **1** and δ_C_ 20.4, δ_H_ 1.99 in **2**) was used to deduce that the C-3′′/4′′ double bond was in Z configuration. Thus, **1** is a geometric isomer of **2** [4].

Hygrolansamycin C (**3**) was isolated as a yellow powder. The molecular formula was determined to be C_28_H_35_NO_8_ on the HRESIMS data, which is the same as that of **2**. The high similarity between the 1 and 2D NMR spectra of 2 and 3 indicated that they shared the same planar structure. The ROESY spectrum correlation between H-2 and H-4_ax_ (δ_H_ 2.98) indicates that *O*-heterocyclic ring is axial preferred for both H-2 and H-4_ax_ (Fig. 4B) [6, 7]. Furthermore, the 1D NMR chemical shifts of C-14 (δ_C_ 15.3, δ_H_ 1.29 in **2** and δ_C_ 9.9, δ_H_ 0.89 in **3**) were shown relative configuration of C-2, which is opposite in between **2** and **3** [17]. Thus, 3 is a stereoisomer of 1.

Hygrolansamycin D (**4**) was isolated as a yellow powder. The HRESIMS data indicated that the molecular formula of **4** was C_28_H_35_NO_8_. The high similarity between the 1 and 2D NMR spectra of **1** and **4** indicated that they shared the same planar structure. The ROESY spectrum (Fig 4B) and chemical shifts of C-14 (δ_C_ 15.5, δ_H_ 1.28 in **1** and δ_C_ 9.9, δ_H_ 0.92 in **4**) were shown configuration of C-2, which is opposite in between **1** and **4**. Furthermore the spectroscopic data of **4** were different the upfield shift of H-11 (δ_H_ = 4.06) and downfield shift of H-12 (δ_H_ = 4.87). The different absolute configuration of **4** was established by the same method as that of **1**. The Δδ [δ(S) - δ(R)] values of the MPA esters indicate the R-configuration at C-11 in **4** (Figs. 4C and 4E). Therefore, the absolute configuration of **4** was established 1S/2S/5S/8R/11R/12R.

The *O*-heterocyclic ring moiety of **5** [7] was present in the four hygrolansamycin congeners **1-4**. However, closer examination of the NMR data revealed some variations at position C-2 in the *O*-heterocyclic ring. The H-1 signals appeared as doublets in the ^1^H NMR spectra of **3** and **4**, but not in those of **1** and **2** [7, 17]. Therefore, H-1 and H-2 are anti orientated in **1** and **2**, while in **3** and **4**, they are *syn*. Furthermore, the C-3′′/C-4′′ double bond shifted to the β, γ-position, rather than the typical α, β-position, which results from traditional *syn* elimination of water from a β-hydroxy function. From the relatively shielded chemical shifts of C-6′′ (δ_C_ 13.1 in **1** and δ_C_ 13.2 in **4**) of compounds **1, 4**, and **5**, C-3′′/4′′ was *E* configuration, whereas the C-6′′ (δ_C_ 20.4) of **2** and **3**, C-3′′/4′′ was determined to be Z configuration [6]. Except for the C-3"/4" double bond, other chromophores have little effect on the Cotton effects. Compounds **1, 4**, and **5** with E forms show different Cotton effects from compounds 2 and 3 in the 200-280 nm wavelength range, but all spectra are very similar at wavelengths above 290 nm. In addition, reported divergolides E, G, H, and O (**5**) show a positive Cotton effects at 260 nm, while **2** and **3** with the Z form show a lower negative Cotton effects at 230-260 nm, similar to those of reported divergolides A and F (Fig. **5**) [4, 6, 9]. Although the *O*-heterocyclic moiety, C-3′′/4′′ double bond and isobutyl moiety of divergolides may serve as additional chromophores, the hygrolansamycins found in this study showed similar CD spectral patterns. These results show that the differences in chromophore of *O*-heterocyclic ring moiety does not affect the CD spectrum patterns of compounds.

Many divergolides and hygrocins are cytotoxic against several cancer cell lines [3-5, 7]. The cytotoxicity of **1-6** was evaluated against five cancer cell lines, namely human gastric adenocarcinoma (AGS), murine melanoma (B16F10), human cervical carcinoma (HeLa), human breast cancer (MDA-MB-231), and human pheochromocytoma (PC12), employing the EZ-Cytox cell viability assay (Table 2). Only compound **2** exhibited moderate cytotoxic activity against the five human cancer cell lines due to the influence of the three-dimensional structure affecting the different Cotton effects (Fig. 5), with other compounds being inactive even at a concentration of 100 μM.

## Figures and Tables

**Table 1:** ^1^H (800 MHz) and ^13^C (200 MHz) NMR data for hygrolansamycins A-D (1‒4) in DMSO-d_6_.

No.	Hygolansamycin A (**1**)		Hygolansamycin B (**2**)		Hygolansamycin C (**3**)		Hygolansamycin D (**4**)	
			
	δ_C_	δ_H_ (mult., J in Hz)	δ_C_	δ_H_ (mult., J in Hz)	δ_C_	δ_H_ (mult., J in Hz)	δ_C_	δ_H_ (mult., J in Hz)
1	74.9	5.04 (m^a^)	74.8	5.02 (m^a^)	75.3	5.16 (d, 5.1)	75.3	5.19 (d, 5.2)
2	51.5	2.45 (dd, 14.5, 7.1)	51.5	2.45 (dd, 14.5, 7.2)	48.7	3.15 (m^a^)	48.6	3.13 (m^a^)
3	208.3		208.4		206.7		206.8	
4	48.4	3.08 (d, 16.7), 2.12 (d, 16.7)	48.6	3.08 (d, 16.7), 2.30 (d, 16.7)	52.1	2.98 (d, 15.3), 2.45 (d, 15.2)	51.3	2.93 (d, 15.2), 2.41 (d, 15.2)
5	102.1		102.0		103.6		103.1	
6	38.1	1.81 (m^a^), 1.58 (t, 12.6)	37.9	1.94 (t, 12.3), 1.87 (m^a^)	37.9	1.94 (m^a^), 1.84 (m^a^)	39.2	1.94 (t, 13.2), 1.79 (t, 13.2)
7	27.9	1.67 (m^a^), 1.22 (m^a^)	28.5	1.68 (td, 12.0, 6.5), 1.08 (d, 6.3)	27.5	1.66 (m^a^), 1.00 (m^a^)	26.3	1.59 (t, 13.0), 1.39 (m^a^)
8	43.5	1.77 (m^a^)	43.7	1.75 (dt, 14.2, 9.3)	43.7	1.76 (m^a^)	44.1	1.84 (m^a^)
9	136.3	5.30 (dd,15.5, 9.4)	134.9	4.94 (ddd, 15.7, 9.1, 1.3)	134.8	4.92 (dd, 15.4, 9.2)	136.4	5.28 (dd, 15.3, 8.5)
10	128.0	5.63 (dd, 15.8, 4.8)	124.7	5.51 (dd, 15.7, 3.8)	124.6	5.51 (dd, 15.7, 3.5)	129.8	5.41 (dd, 15.7=6, 7.3)
11	77.3	5.02 (ddd, 6.8, 4.8, 1.0)	78.2	5.04 (m^a^)	78.2	5.01 (m^a^)	72.0	4.06 (dd, 7.0, 3.4)
12	68.5	3.77 (qd, 6.8, 6.5)	67.5	3.72 (dq, 6.8, 7.0)	67.4	3.77 (m^a^)	73.0	4.87 (dd, 6.0, 3.8)
13	19.2	1.08 (d, 6.4)	18.8	1.08 (d, 6.3)	18.8	1.07 (d, 6.2)	13.8	1.17 (d, 6.4)
14	15.5	1.28 (d, 7.2)	15.3	1.29 (d, 7.2)	9.9	0.89 (d, 6.6)	9.9	0.92 (d, 6.8)
15	28.8	1.37 (m^a^), 1.22 (m^a^)	27.6	1.37 (qd, 12.6, 7.2), 1.29 (m^a^)	28.5	1.35 (dd, 12.9, 7.0), 1.27 (dd, 15.0, 6.9)	28.1	1.39 (m^a^), 1.25 (tt, 14.7, 7.2)
16	12.4	0.83 (t, 7.4)	12.5	0.83 (t, 7.4)	12.5	0.82 (t, 7.4)	12.1	0.83 (t, 7.4)
1′	106.6	6.22 (d, 2.7)	105.9	6.16 (d, 2.7)	107.3	6.06 (d, 2.5)	110.2	6.21 (d, 2.1)
2′	151.2		151.2		150.0		149.8	
3′	107.7	7.51 (d, 2.7)	106.9	7.68 (d, 2.7)	107.6	7.69 (d, 2.5)	111.9	6.91 (d, 2.0)
4′	126.4		127.6		127.2		126.0	
5′	132.6		131.7		132.0		135.8	
6′	122.5		122.4		119.3		120.0	
1″	166.6		168.2		168.2		168.0	
2″	37.5	3.37 (dd, 16.6, 8.1), 3.29 (dd, 16.6, 8.0)	39.9	4.06 (t, 11.6), 2.85 (dd, 11.3, 7.2)	39.8	4.07(t, 11.6), 2.86 (dd, 11.4, 7.1)	37.7	3.31 (dd, 14.2, 7.3), 3.13 (ma)
3″	133.4	7.00 (td, 7.9, 1.1)	136.7	6.29 (dd, 11.4, 7.0)	136.8	6.29 (dd, 11.4, 7.1)	135.2	6.75 (t, 6.2)
4″	133.9		138.4		134.1		130.9	
5″	166.4		166.6		166.5		166.5	
6″	13.1	1.86 (s)	20.4	1.99 (s)	20.4	1.99 (s)	13.2	1.84 (s)
NH		7.97 (s)		8.82 (s)		8.77 (s)		8.90 (s)
2′-OH		9.15 (s)		9.08 (s)		9.02 (s)		9.05 (s)

aResonances overlapped or multiplet.

**Table 2:** Cytotoxicity of 1‒6 (IC_50_: μM)

Compound	AGS	B16F10	HeLa	MDA-MB-231	PC12

doxorubicin	1.37 ± 0.44	1.00 ± 0.06	0.82 ± 0.03	1.83 ± 0.07	3.48 ± 0.02
**1**	>100	>100	>100	>100	>100
**2**	24.60 ± 3.37	49.93 ± 4.52	41.45 ± 0.36	46.71 ± 2.22	34.08 ± 0.35
**3**	>100	>100	>100	>100	>100
**4**	>100	>100	>100	>100	>100
**5**	>100	>100	>100	>100	>100
**6**	>100	>100	48.97 ± 2.49	>100	>100

Cell lines are human gastric adenocarcinoma (AGS), murine melanoma (B16F10), human cervical carcinoma (HeLa), human breast cancer (MDA-MB-231), and human pheochromocytoma (PC12) cell line.
